# A novel *GPR143* splicing mutation in a Chinese family with X-linked congenital nystagmus

**Published:** 2011-03-12

**Authors:** Junjie Hu, Desheng Liang, Jinjie Xue, Jing Liu, Lingqian Wu

**Affiliations:** State Key Laboratory of Medical Genetics, Xiangya Hospital, Central South University, Changsha, Hunan, China

## Abstract

**Purpose:**

The purpose of the current research was to detect the underlying genetic defect in a Chinese family with X-linked congenital nystagmus and perform prenatal genetic diagnosis for their current pregnancy.

**Methods:**

A common clinical examination and an ophthalmic evaluation were performed on the proband, one carrier, and one unaffected member. Mutation analysis of the G protein-coupled receptor 143 (*GPR143*) and four-point-one (4.1), ezrin, radixin, moesin (FERM) domain-containing 7 (*FRMD7*) genes was performed by direct sequencing of PCR-amplified exons in the proband. The detected *GPR143* mutation was tested in all available family members and 200 normal controls by direct sequencing.

**Results:**

Congenital nystagmus, obvious fundus hypopigmentation, and foveal hypoplasia were observed in the proband but not in the carriers or the unaffected members. A novel splicing mutation c.658+1 g>t not found in 200 unrelated controls was identified and co-segregated with X-linked ocular albinism (XLOA) in this family. The fetus (V:5) was hemizygous for this mutant allele.

**Conclusions:**

We identified a novel causative mutation of *GPR143* in a five-generation Chinese family with XLOA. This expanded the mutation spectrum of *GPR143* and provided data elucidating the diverse and variable effects of *GPR143* mutations.

## Introduction

Congenital nystagmus (CN) is a common oculomotor disorder with a frequency of 1/20,000 live births [1]. It is characterized by involuntary, rhythmical, repeated oscillations of one or both eyes with onset typically at birth or within the first few months of life. Patients’ oscillations can be horizontal, vertical, torsional, or any combination of these, although horizontal is the most common [2].

CN is genetically heterogeneous. Two genes responsible for X-linked CN have been identified (i.e., G protein-coupled receptor 143 (*GPR143*) for X-linked ocular albinism

(XLOA) [3] and four-point-one (4.1), ezrin, radixin, moesin (FERM) domain-containing 7 (*FRMD7*) for X-linked CN-1 [4,5]). The third X-linked form of CN has been mapped to Xp11.4-p11.3 [6].

Here, we report on a five-generation Chinese family with X-linked CN. Mutation analysis of the two candidate genes (*FRMD7* and *GPR143*) indicated that a novel splicing mutation of *GPR143* was responsible for the disease in this family.

## Methods

### Patients

**Figure 1:**
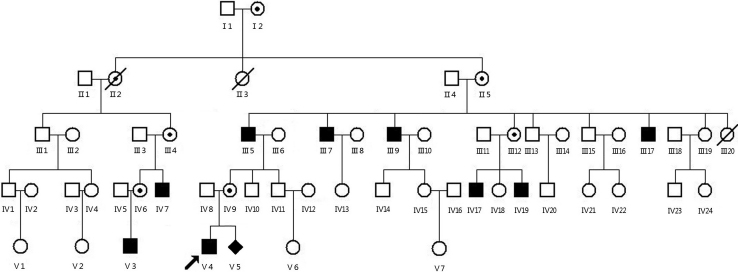
The Chinese CN pedigree. Black-filled symbols indicate patients who carried the novel mutation in the hemizygous state. Dot-marked symbols represent females who carried the mutation. The proband is marked by arrow.

The CN family studied here came from the Hunan province in the southern part of China and comprised five generations with nine affected members (Figure 1). The proband (V:4) was referred to our clinic at the age of seven because of his horizontal CN in both eyes. After an uneventful 38-week pregnancy of the primigravid mother, he was born at a normal weight. Consanguinity was denied, and no chemical exposure or maternal drug use was noted during his mother’s pregnancy. This study complied fully with the Tenets of the Declaration of Helsinki, and it was approved by the Ethics Board of the State Key Laboratory of Medical Genetics of China. Informed consent was given by all members of the family before testing.

### Mutation screening

Genomic DNA was extracted from the peripheral blood using the standard phenol/chloroform method and stored at −20 °C. Mutation analysis of *GPR143* and *FRMD7* was performed using polymerase chain reaction (PCR) and direct sequencing. Primers were designed to cover the sequences of all exons and introns adjacent to each exon of *GPR143* and *FRMD7*, according to published primer sequences (Table 1) [7,8]. PCR products were directly sequenced with an ABI PRISM BigDye kit (Carsbad, CA) using the same ampliﬁcation primers on an ABI3100 DNA sequencer (Carsbad, CA). Sequences were analyzed with the DNASTAR (Madison, WI) software.

**Table 1:** Primers and PCR conditions used to amplify genomic segments of *GPR143* and *FRMD7*.

**Primer name**	**Forward primer(5’-3’)**	**Reverse primer(5’-3’)**	**Annealing temperature (°C)**	**Product size (bp)**
*GPR143*-1a	CTCCTCCGCCCGCCCAAGCATCAC	CCCAGGCAGCCGAGAAGGTC	66	464
*GPR143*-1b	CCGCGCCTAGGGACCTTCTGCT	AACCCGCGGGCCTCTCGTCCTCAC	69	399
*GPR143*-2	CTTTCTTCCTTTTCCCTCCTTGTC	GTTTGCTGCTGCTGCGATTTG	61	360
*GPR143*-3	CACGTGCGGCTTCCTGAC	TTGGCCTCTTATAAAAATGA	59	385
*GPR143*-4	GGGCTTTCCTCTGTGTACATTTTC	CCCTGAGACAACGGCCTAACC	63	334
*GPR143*-5	GCATTTCCCTTTTTGTTCTCATCC	AGGCCTGCACATTTTCATTTATTG	61	406
*GPR143*-6	TTGCTTCCTGCCCCTCTGG	ACTTGCTCCCCTGTCCTCTGT	63	400
*GPR143*-7	TGCACCTGGCCCTCTTAGTTTC	TCAGGAGGCCAAGACAGAGGAT	63	441
*GPR143*-8a	AAACCAACCCACCAACCAGTCAAC	GCATGCTCAGGGCTTCGTCA	63	395
*GPR143*-8b	CCAGCCCAGGGATTTCTCTT	ACCCCGCCATGCACAGGAC	63	329
*GPR143*-9	AGCTGATGACAAACCTGCTAG	CCCTTTCTCCTATCCTAAAG	61	330
*FRMD7*-1	CCTTGGGTGTGCATTACTTC	TTTGCTATTGTTGTCCCTTGAG	57	459
*FRMD7*-2	AAACAACACAGAGACAGATAAGTGG	CAATCAGGGAATTGAACCCTAC	57	385
*FRMD7*-3	AGGCAGTGGAGCAGTGATTC	GCAGCATGATTTCTTTCATCTC	68	499
*FRMD7*-4	CTCGAAGGCAGAGAGGGTAG	CCCTTTGGATGATGAACACC	69	519
*FRMD7*-5	GGCACCATTCCTTTCTTGAAT	CAGGCCATGCTGTTTCTCTC	57	350
*FRMD7*-6	TTTGGACTGCATTGGCTACA	AGGATCTCAGCGTTTCATGG	57	353
*FRMD7*-7	TCATGCACTTTCATCAGAAGC	TGATTGACCATTTCCCTTTC	57	497
*FRMD7*-8	TGTGCAAGAGATGGGTCAAG	CTCTGGTTGATTTCTTCAAAGG	57	368
*FRMD7*-9	GCTCTGTTTGTGAGCAGTGG	AGGGTGCAATCTTTGATGTG	57	495
*FRMD7*-10	AGGTTGTTCTCTGCCTGGTC	GCACTGTCGTTCATGGTACTG	57	398
*FRMD7*-11	TGTTTCTCTTGCTCGTGTTGA	TTTTTACACACTGGGATTCTGG	57	282
*FRMD7*-12a	CCCTAGAATAGAACATGGATCTTG	TGGGATCAGGGTTAGGATTG	57	388
*FRMD7*-12b	CCTTCTTTCACCAATGTGTCC	AATACCAACCTGCTGACCTG	57	452
*FRMD7*-12c	CTTTAACACTGAGCCCAATC	TGACTGAGAGCAGGACAAGG	57	588
*FRMD7*-12d	ACGGATGTGCCCTATATTCC	GCAACTCCTGCTCTGCAAAC	57	472
*FRMD7*-12e	AGCCCAAGGAATATCAGAATG	GCAGTTGGTGTGTTGAAATAAGC	57	500
*FRMD7*-12f	GCTCTCAGTCATAAAGCAGACC	CCTTCAGAGGTAATGGAAGAGTG	57	500

A total of 200 healthy, unrelated volunteers were recruited as controls, including 103 males and 97 females aged 10–60 years with an average age of 33. The novel splicing mutation of *GPR143* was tested in the 200 healthy controls by direct sequencing.

## Results

### Clinical findings

Nine males in three successive generations of the Chinese family were affected, indicating that the disease may be inherited in an X-linked recessive pattern in this family (Figure 1). The proband presented with typical symptoms of CN, including nystagmus, amblyopia, foveal hypoplasia, and fundus hypopigmentation. Results of a computed tomography (CT) scan of the brain were normal in the proband.

#### Nystagmus and reduced visual acuity

The nine patients all had nystagmus and poor visual acuity as a first symptom. The nystagmus was present during their first three months of life with a corrected visual acuity (VA) of 0.1–0.2. The proband’s (V:4) nystagmus was sometimes associated with head nodding, and the amplitude varied with horizontal gaze position. In all patients, the nystagmus had a tendency to diminish with age, while it rarely disappeared completely (e.g., the proband’s grandfather (III:5) continued to experience nystagmus even at age 60). The proband’s mother (IV:9), a carrier, did not experience nystagmus, and her VA was normal.

#### Fundus hypopigmentation and foveal hypoplasia

**Figure 2:**
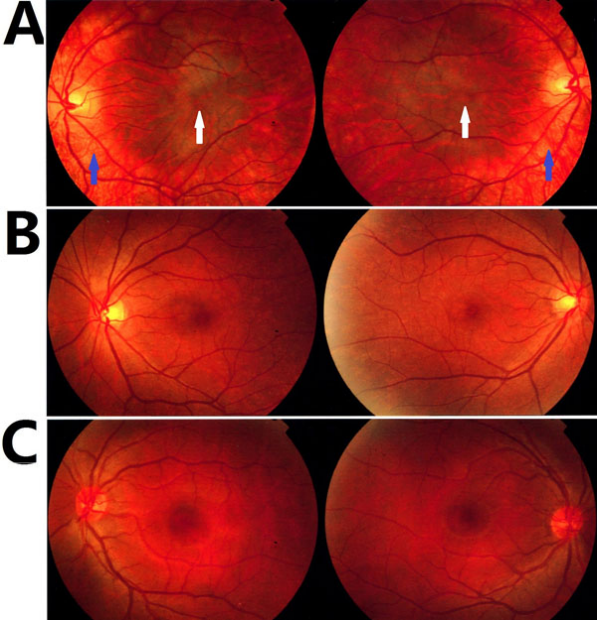
Fundi photographs. **A**: Fundus of the proband (V4) revealed severe fundus hypopigmentation (blue arrow) and foveal hypoplasia (white arrow). **B**: The fundus of the carrier mother (IV9). **C**: Normal fundus (IV10).

In the present study, fundus examinations were performed in most patients, carriers, and unaffected members of the family (Table 2). Significant hypopigmentation of the ocular fundus periphery with normal pigmentation of skin and hair and foveal hypoplasia were observed in the proband (Figure 2A), while his mother (Figure 2B) and his uncle (Figure 2C) did not exhibit such symptoms.

**Table 2:** Summary of clinical features of some affected males and carriers.

**ID# patients**	**Gender**	**Iris hypopigmentation**	**Albinotic fundus**	**Fundus hypopigmentation**	**Fundus foveal hypoplasia**	**Nystagmus**
V:4	Male	Mild	No	Obvious	Obvious	Yes
III:5	Male	Mild	No	Obvious	Obvious	Yes
III:7	Male	Mild	No	Obvious	Obvious	Yes
III:9	Male	Mild	No	Obvious	Obvious	Yes
III:17	Male	Mild	No	Mild	Obvious	Yes
IV:17	Male	Obvious	No	Obvious	Obvious	Yes
**Carriers**						
IV:9	Female	Normal	No	Normal	No	No
III:12	Female	Normal	No	Normal	No	No
II:5	Female	Normal	No	Normal	No	No

#### Iris hypopigmentation

**Figure 3:**
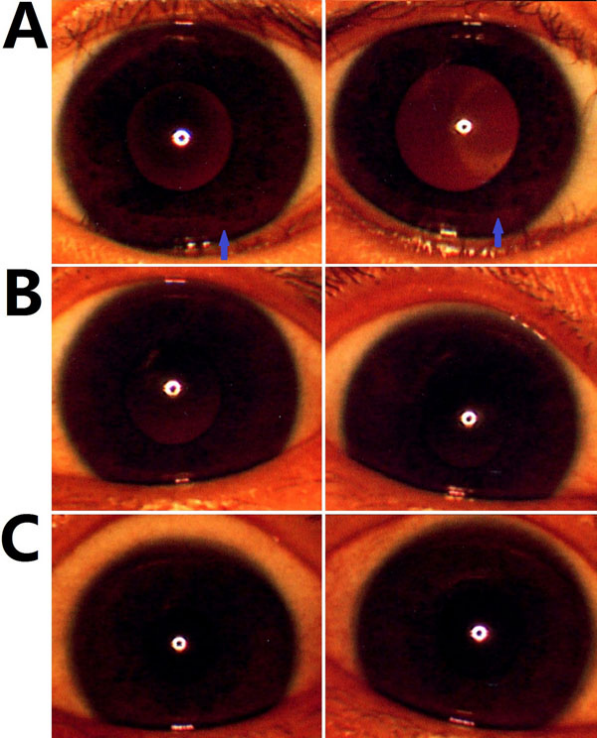
Iris photographs. **A**: Irises of the proband (V4) revealed mild hypopigmentation (blue arrow). **B**: Irises of the carrier mother (IV9). **C**: Normal irises of an unaffected member (IV10).

Compared with an unaffected member (Figure 3C), the proband exhibited mild peripheral iris hypopigmentation, as shown in Figure 3A. As a carrier, his mother did not show peripheral iris hypopigmentation (Figure 3B).

### Mutation screening

**Figure 4:**
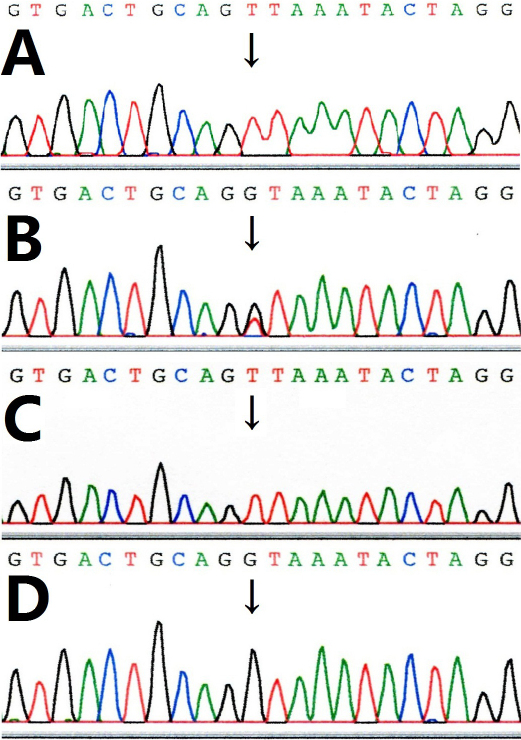
Sequencing of GPR143. **A**: Sequence in the proband (V4) showing a novel splicing mutation c.658+1G>T. **B**: Sequence in the proband's mother (IV9) revealing a heterozygous mutation. **C**: The sequencing result of the fetus (V5) hemizygous for the mutant allele. **D**: Sequence in an unaffected male member (III6) hemizygous for the wild type allele.

The nine patients and the fetus (V:5) were hemizygous for a novel splicing mutation c.658+1G>T (Figure 4A,C), while carriers were heterozygous for this genotype (Figure 4B) and unaffected members were normal (Figure 4D). The splicing mutation c.658+1G>T was not found in the 200 unrelated controls.

## Discussion

In 1995, Schiaffino et al. [3] screened the entire *GPR143* coding sequence and detected various mutations in one-third of XLOA patients. To date, more than 99 different mutations of *GPR143*, involved in most exons, have been published [9]. Prior to 2007, XLOA in the Chinese population was infrequently reported [2], and to date, only 11 mutations of *GPR143* have been described in the Chinese population including 3 missense mutations [2,7,10], 1 splicing mutation [7], 6 deletion mutations [7,11,12], and 1 duplication mutation [13]. The 11 mutations were all associated with nystagmus but without ocular albinism (OA). In the present study, the novel mutations of c.658+1G>T were found to cause CN in a large Chinese family. The cumulated number and ethnic distribution of known mutations will help further study in the pathogenesis of CN.

*GPR143* on chromosome Xp22.3 contains nine exons and encodes a protein of 404 amino acids containing seven putative transmembrane domains and one potential N-glycosylation site using an asparagine at codon 106 [14]. *GPR143* is mainly expressed in skin and retinal pigmented epithelial cells. The GPR143 protein is a G protein-coupled receptor (GPCR) that is embedded in the melanosome membrane [15], with the NH_2_-terminus of the protein in the melanosome lumen and the COOH-terminus in the cytosol.

**Figure 5:**
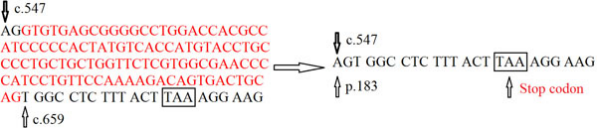
Schematic diagram showing a possible result from the novel mutation of GPR143: When the original splicing donor disappears, the exon 5 (bases in red font) is lost, hence introducing a new stop codon and creating a truncated protein of 187 amino acids.

In the present study, we found a splice site mutation of *GPR143* at the exon-intron 5 boundary (c.658+1G>T) and in the 5′ consensus donor region for the splicing of intron 5–6. This mutation may lead to two possible effects: the loss of the original splicing donor or the generation of a new splice site. If the original splicing donor disappears, the exon 5 of *GPR143* may be lost, thus leading to the introduction of a stop codon. This creates a truncated protein of 187 amino acids (Figure 5), which is much shorter than the normal full-length protein of 404 amino acids, and it may seriously affect the function of this protein. On the other hand, we searched for potential abnormal splice sites generated by this mutation using NNSPLICE software [16] and found some possible displaced splice sites. Being used instead of the missing original splice site, the neighboring cryptic splice sites might result in short, erratic sequences ending with a stop codon after the normal sequence. If the abnormal mRNA is actually translated and escapes degradation, the encoded protein will be truncated and dysfunctional.

XLOA, a disorder of melanosome biogenesis leading to congenital and persistent visual impairment in affected males, is characterized by CN, reduced visual acuity, hypopigmentation of the iris pigment epithelium and the ocular fundus, and foveal hypoplasia [17]. XLOA is a non-progressive disorder, and visual acuity remains stable throughout life. Nystagmus has been reported in ocular albinism patients with mutations of *GPR143* and is thought to be a secondary phenotype in these patients [18]. However, one of the classical OA phenotypes, ocular albinism, has rarely been observed in patients with *GPR143* mutations. Fang et al. [7] found a similar splicing mutation c.658+1G>A in a family with XLOA; however, the patients’ phenotypes differed from those of our patients. While fundus hypopigmentation existed in our proband but not in theirs, both showed mild iris hypopigmentation. On the contrary, fundus hypopigmentation appeared in their carriers but not in ours. It is still unclear why these two mutations at the same locus (c.658+1G>T and c.658+1G>A) in *GPR143* cause different phenotypes. It is possible that the new splice site generated by these two mutations will result in the expression of part intron in the 5′ consensus donor region for the splicing of intron 5–6. Thus, the mutation c.658+1G>T will introduce a new amino acid p.220Val, while c.658+1G>A will introduce p.220Asp. Different amino acids may lead to different protein structures and eventually produce different phenotypes.

The role of *GPR143* in the development of the visual system is currently poorly understood [19]. In the present study, we identified a novel causative mutation of *GPR143* and offered a reliable prenatal genetic diagnosis in a five-generation Chinese family with XLOA. Our findings both expand the mutation spectrum of *GPR143* and provide data elucidating the diverse and variable effects of *GPR143* mutations.
